# Room Temperature Ferromagnetism in InGaN Nanostructures Induced by Cr^+^ ion Implantation

**DOI:** 10.3390/nano10061128

**Published:** 2020-06-08

**Authors:** Zheng Wang, Hao Wu, Yong Liu, Chang Liu

**Affiliations:** 1 Key Laboratory of Artificial Micro- and Nano-structures of Ministry of Education, and School of Physics and Technology, Wuhan University, Wuhan 430072, China; wz@whu.edu.cn; 2 Hubei Nuclear Solid Physics Key Laboratory, and School of Physics and Technology, Wuhan University, Wuhan 430072, China; yongliu@whu.edu.cn

**Keywords:** ions implantation, molecular beam epitaxy, nitrides, magnetic materials

## Abstract

This paper presents the magnetic properties of chrome ion (Cr^+^) implanted In_x_Ga_1−x_N (x = 0.1, 0.3, 0.5 and 1.0) nanostructures grown by molecular beam epitaxy (MBE). The Cr^+^ implantation was conducted at 110 keV with three doses, namely 2.6 × 10^15^, 5.3 × 10^15^, and 1.3 × 10^16^ ions/cm^2^. The as-grown nanostructures exhibited diamagnetism before and after ion implantation without annealing. However, after annealing, the nanostructures exhibited ferromagnetism at room temperature. The saturation magnetization (Ms) and coercive force (Hc) increase with increasing Cr^+^ dose. The Ms of the InN nanorods with diameters of 100–160 nm is larger than that of those with small diameters of 60–80 nm. For InGaN nanostructures, the indium concentration—that is, the band structure—is more important than the diameters of the nanorods for the same doping level of Cr ions. The Ms of InGaN nanorods with an indium concentration of 10% reaches its maximum. The zero-field cooled (ZFC) and field-cooled (FC) curves show that nanostructures have no parasitic magnetic phases.

## 1. Introduction

The study of ferromagnetism in diluted magnetic semiconductors (DMSs) with Curie temperature (T_C_) significantly above room temperature has gained great attention due to their potential applications in spintronic devices [1,2]. According to the theoretical predicting [3,4], transition metals (TM) such as V, Cr, Mn, Fe, Co, and Ni that have partially filled d states can be doped into III–V and II–VI semiconductors transforming them into ferromagnets. There have been a number of experimental results on room-temperature ferromagnetism in transition metal (Mn,Cr) doped GaN and InN thin films [5,6,7,8,9]. However, research on ferromagnetism on InGaN nanostructures is still rare [10,11]. InGaN has the accessibility of band gap modulation (from 0.7 eV for InN to 3.4 eV for GaN), which carries a high potential for wide-ranging applications.

The doping concentration of the transition metals in semiconductors is an important factor affecting the magnetic properties of the DMS. Therefore, it is important to study the effects of various doping concentrations on the magnetic properties. Most studies on the ferromagnetism of Cr doping [5,6,7] were based on Cr concentrations greater than 3%. In-situ growth is difficult to accurately control the amount of Cr doping at low concentrations according to our early work [12,13]. Cr doping also has a negative effect on the growth of InGaN nanorod structures. Ion implantation technology can precisely control the total dose of impurities and the dopant distribution along depth. Batch processing samples by one implantation is also possible, showing great advantages to implant the same dopants into different substrates.

Recently, defect-induced room temperature ferromagnetism in GaN films [14], InN films [15], and InN nanorods [16] was reported. Theoretical investigations suggested that gallium vacancy (V_Ga_) is the source of ferromagnetic properties in undoped GaN [17]. Further calculations [18] suggest that V_Ga_ cannot induce room temperature ferromagnetism at a density lower than 10^21^ cm^−3^. In fact, there are many room temperature ferromagnetic results, but the defect densities of the samples are not described in detail. Therefore, the effect of defects on ferromagnetism needs further study. For ion implantation technology, defects were introduced when implanting TMs; afterwards, annealing can reduce defects and drive the dopants into the lattice sites [19,20]. The effects of defects and TM doping on ferromagnetism can be well studied by ion implantation.

In this work, we grew InN nanostructures on GaN substrates and InGaN nanorods on Si (111) substrates with various indium concentrations using MBE. We used the self-assembly growth method to prepare samples to avoid the damage to the crystalline quality of the sample caused by etching during the patterning process [21]. The Cr^+^ were homogeneously implanted into the samples at an energy of 110 keV with three doses, corresponding Cr concentrations of 0.5%, 1.0%, and 2.5%, respectively. The effects of Cr dopants and defects on InN samples with different morphologies were discussed through experiments. On this basis, the effect of indium concentration of InGaN nanorods on magnetic properties was studied.

## 2. Results and Discussions

**Figure 1:**
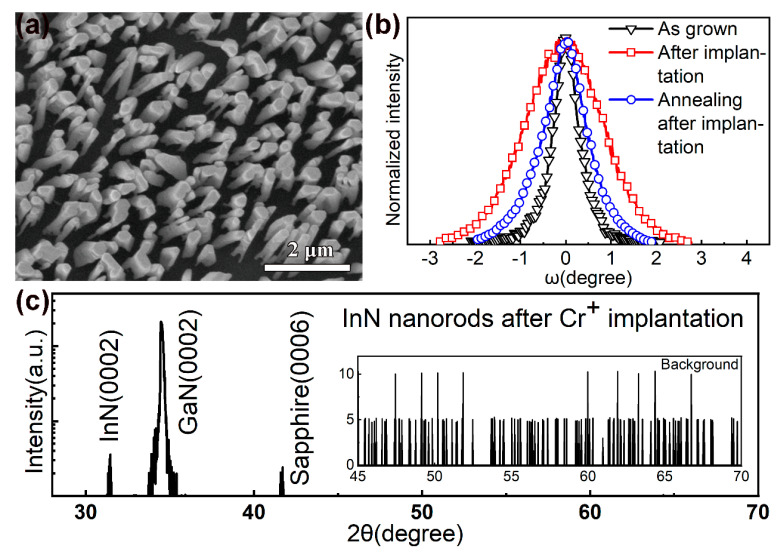
(**a**) Field-emission scanning electron microscope (FESEM) image of the InN nanorods on GaN (0002) substrates; (**b**) (0002) rocking curves of the InN nanorods; (**c**) XRD θ–2θ scans of the InN nanorods.

After the Cr^+^ implantation, the InN nanorods still maintain the wurtzite structure. Figure 1a shows the field-emission scanning electron microscope (FESEM) image of the InN nanorods on GaN (0002) substrates. Figure 1b shows the (0002) rocking curves of the InN nanorods. The implantation damaged the InN nanorods, increasing the full width at half maximum (FWHM) from 0.7° to 2.0°. Annealing effectively removed the damage and reduced the FWHM to 1.1°, but the damage did not fully recover. Figure 1c shows the X-ray diffraction (XRD) 2θ-ω patterns of the InN nanorods after the implantation. The diffraction peaks at 31.4° and 34.6° correspond to the hexagonal wurtzite-type InN (0002) and GaN (0002). No ferromagnetic impurity phase was observed in the XRD data.

**Figure 2:**
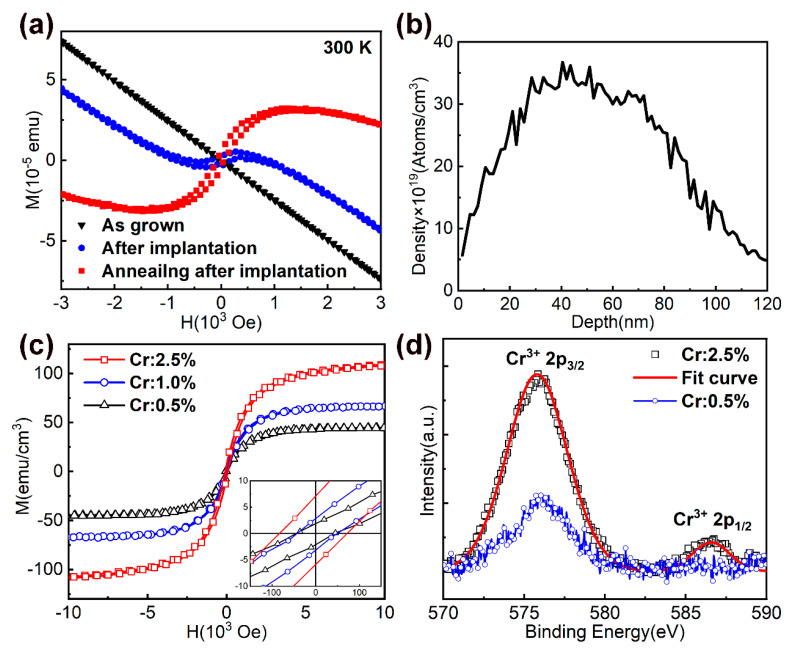
(**a**) Magnetic properties of as-grown InN nanorods and the InN nanorods after implantation (Cr: 2.5%) and the InN nanorods annealing after implantation; (**b**) SRIM simulation of depth distribution of 110 keV Cr^+^ ions with dose of 1.3 × 10^16^ ions/cm^2^; (**c**) M–H curves for the InN nanorods with different doses of Cr^+^ implantation (Cr: 0.5%, Cr: 1.0%, Cr: 2.5%). Inset shows hysteresis at lower fields; (**d**) XPS spectra of the implanted InN nanorods.

The as-grown nanostructures exhibited diamagnetism before implantation. After implantation, most nanostructures still exhibited diamagnetism, and only a few samples with Cr:2.5% exhibited a mixture of diamagnetic and ferromagnetic behavior at room temperature, as shown in Figure 2a. In addition, no AFM transition was found in the as-implanted samples at low temperature. After annealing, most nanostructures exhibit a mixture of diamagnetic and ferromagnetic behaviors at room temperature. Figure 2b shows the Stopping and Range of Ions in Matter (SRIM) simulation of depth distribution of 110 keV Cr^+^ ions with a dose of 1.3×10^16^ ions/cm^2^ and corresponding Cr concentration of 2.5%. The Cr concentration appearing in this article refers to the doping concentration calculated during ion implantation, and it does not represent the exact Cr concentration in the samples. It has also to be noted that the experimental uncertainty to determine the effective magnetic moment depends on the volumes of the samples. According to the relationship between implantation depth and implantation dose, as shown in Figure 2b, we set the sample height to 120 nm. When the magnetic contributions from the GaN layer and sapphire substrates or Si substrates with a buffer layer are subtracted out, the M–H data show a clear hysteresis loop, indicating a characteristic ferromagnetic behavior, as shown in Figure 2c. According to the literature [14,15,16,17,18], a high concentration of cationic vacancies can induce room temperature ferromagnetism. When Cr^+^ ions were implanted, cation vacancies were introduced. As the Cr^+^ dose increased, the cation vacancies increased, but most as-implanted samples were not found to be ferromagnetic. Although annealing reduced the cation vacancies, the annealed samples showed ferromagnetism, indicating that the doped Cr played a major role. After annealing, the Ms and Hc increased with increasing Cr^+^ dose. For InN nanorods, the Cr^+^ dose increased from 0.5% to 1.0%, the change of Hc was negligible, but the change of Ms was obvious. When the Cr^+^ dose was increased to 2.5%, Hc and Ms increased significantly. The results are shown in Figure 2c.

After the implantation, Cr could occupy either the interstitial sites or substitute In or In vacancies in the InN lattice. Some simulation results [17,22,23] show that Cr atoms prefer to occupy Ga sites in GaN and have a strong tendency to form embedded clusters while maintaining the wurtzite structure. For Cr clusters larger than 2 atoms, antiferromagnetic (AFM) states are energetically preferred over ferromagnetic (FM) and nonmagnetic states. As annealing can drive the dopants into the lattice sites, the appearance of ferromagnetism should be related to the Cr occupying the In sites. In order to exclude the possibility of Cr-rich impurity phases, X-ray photoelectron spectroscopy (XPS) analysis is carried out. Figure 2d shows the XPS spectra of the implanted InN nanorods. For as-implanted samples, XPS results confirmed the presence of Cr, but the valence state can not be characterized. After annealing, Cr^3+^ 2p_3/2_ will appear. When the Cr dose is increased to 2.5%, Cr^3+^ 2p_1/2_ will appear. The solid line is a fitting result indicating the presence of only 3+ valence state after annealing, which indirectly proves that substitution doping has occurred. The optimal dose of Cr implantation depends on how many Cr atoms occupy the substitution positions. In the case of ensuring crystalline quality, the greater the Cr dose, the stronger the Ms of the sample. When the doped material meets the requirements of magnetic properties, the smaller the Cr dose, the better. Here, 3% Cr atomic concentration may be the optimal value.

**Figure 3:**
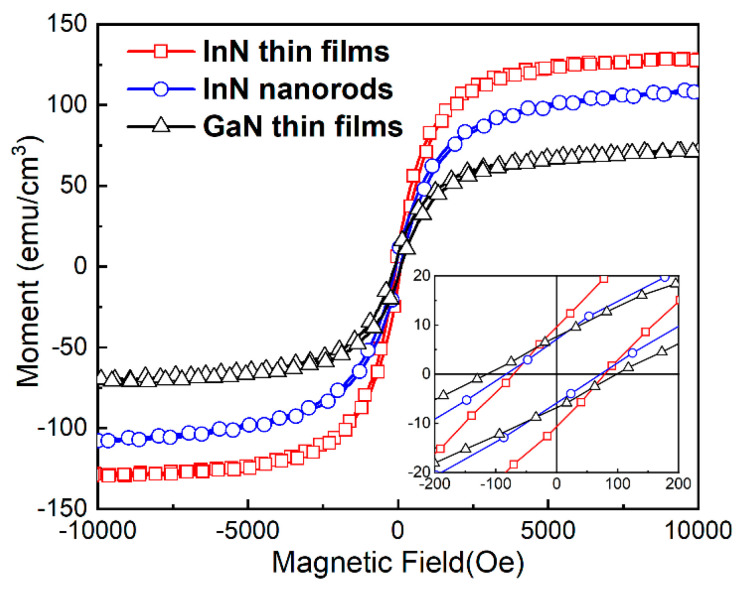
Magnetic properties of InN samples (Cr:2.5%) on GaN substrates at 300 K. Inset shows hysteresis at lower fields.

Comparing the hysteresis loops in Figure 3, it is found that the Ms of the InN nanorods (114 emu/cm^3^) is larger than that of the GaN thin films (70 emu/cm^3^), but smaller than that of the InN thin films (128 emu/cm^3^). Hc of the InN nanorods (75 Oe) is similar to InN thin films (77 Oe), but smaller than that of the GaN thin films (105 Oe). Ms decreases with increasing indium concentration, while Hc increases with increasing indium concentration. GaN has the lowest saturation magnetization and largest Hc due to its wide energy band. In general, the band gap of nanorods is wider than that of thin films, which may be the reason why the Ms of InN nanorods is smaller than that of InN thin films when the Hc is similar.

**Figure 4:**
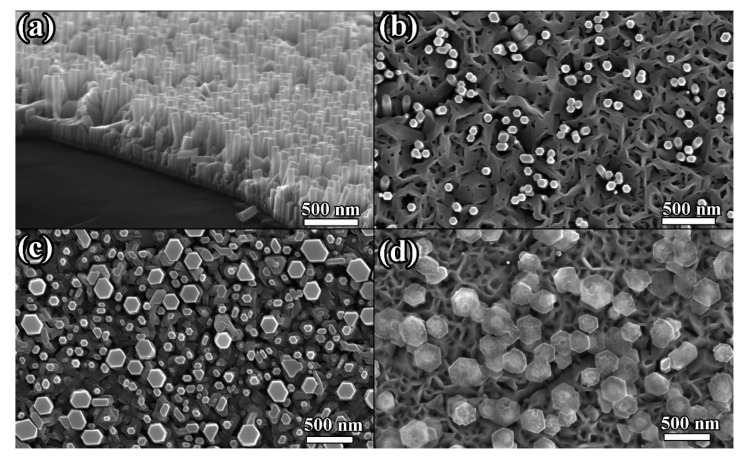
FESEM images of InGaN nanostructures on Si (111) substrates. (**a**) Side view of InGaN nanorods; (**b**) Top view of the In_0.1_Ga_0.9_N nanorods; (**c**) Top view of the In_0.3_Ga_0.7_N nanorods; (**d**) Top view of the In_0.5_Ga_0.5_N nano-mushrooms.

**Figure 5:**
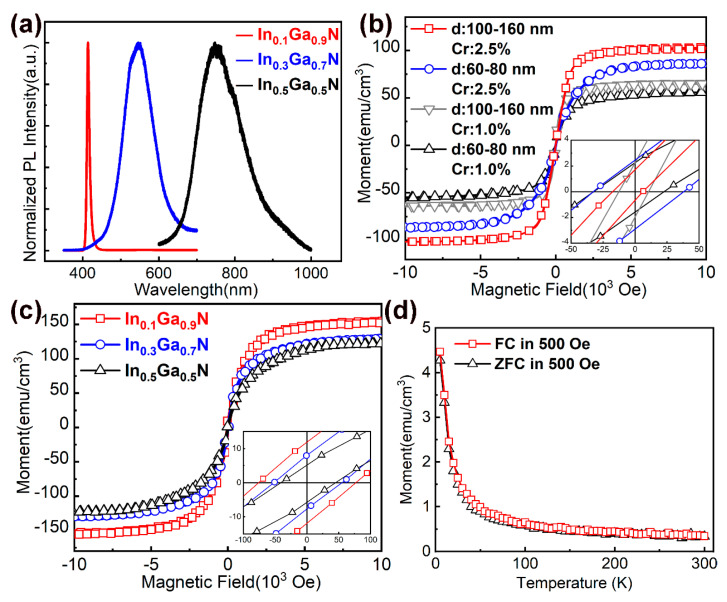
(**a**) Normalized photoluminescence (PL) spectra of In_x_Ga_1-x_N (x = 0.1, 0.3, 0.5) nanostructures; (**b**) Magnetic properties of InN samples (Cr = 1.0% and Cr = 2.5%) with diameters of 60–80 nm and 100–160 nm on Si (111) substrates at 300 K. Inset shows hysteresis at lower fields; (**c**) Magnetic properties of In_0.1_Ga_0.9_N, In_0.3_Ga_0.7_N, and In_0.5_Ga_0.5_N nanostructures on Si (111) substrates at 300 K. Inset shows hysteresis at lower fields. (**d**) Zero field-cooled and field-cooled magnetization curves for the In_0.1_Ga_0.9_N nanorods.

We further studied the effects of nanostructure morphology and indium concentration on the magnetic properties. By regulating the AlN buffer layers on Si (111) substrates, the density and diameter of the nanorods can be controlled. The characterization of the nanorods can refer to our previous work [24]. Figure 4 shows the FESEM images of InGaN nanostructures on Si (111) substrates. As the indium concentration increases, the size of the nanorods increases. After the indium concentration is greater than 20%, the morphology of the nanorods is difficult to control. The diameter of most In_0.1_Ga_0.9_N nanorods is distributed in the range of 70–90 nm. For In_0.3_Ga_0.7_N samples, the diameter of nanorods is distributed in the range of 70–350 nm. Although the diameter range of In_0.3_Ga_0.7_N nanorods is broad, no photoluminescence (PL) peak with a low indium component is observed, as shown in Figure 5a. When the indium is excessive, the morphology of the nanorods can not be maintained. If the indium concentration is greater than 50%, nano-mushrooms form. This is consistent with the report by Morassi et al. [25]. Figure 5b shows the magnetic properties of InN nanorods (Cr: 1.0% and Cr: 2.5%) with diameters of 60–80 nm and 100–160 nm on Si (111) substrates at 300 K. For both doses, InN nanorods with a diameter of 100–160 nm have larger Ms and smaller Hc than those with a diameter of 60–80 nm. For InGaN nanostructures (Cr: 0.5%), it is considered that there is no ferromagnetism after subtracting the contribution of the silicon substrate, which is different from the case on GaN substrates, indicating that a small amount of Cr atoms is not enough to introduce magnetism. Generally, nanorods grown by MBE are considered as single crystals, and the larger the diameter, the larger the crystalline grains and the better the crystallinity. After Cr^+^ implantation, nanostructures with larger sizes may have more Cr occupying the substitution sites. According to the literature [16,26], the defect formation energies at the surface are lower, resulting in a high concentration of defects, which gives rise to percolative ferromagnetism at the surface of the nanoparticles. As the sizes of the particles increase, the defect concentration decreases, and thus, the magnetism vanishes. From our experimental results, in the competition between the two magnetic sources of defects and doping, the doping of Cr is dominant. The defects here refer to the cation (Ga, In) vacancies. The conclusion of the theoretical calculation [17,18] shows that the long-range order is possible among localized moments created by cation vacancies. The magnetic coupling is AFM between neutral cation vacancies but becomes FM upon charging the defect states with electrons. When magnetism is introduced by Cr doping, the coupling between two Cr atoms on the substitution sites could be FM. The FM or AFM state depends on the atomic geometry and the Cr–Cr distances [22,23,27]. However, when the Cr doping concentration is as low as 1.0%, it is difficult for such a small amount of Cr to occupy adjacent lattice sites and form long-range ordered magnetic properties. We believe that the introduction of FM is related to variation of the band structure of nitride semiconductors through Cr^+^ implantation. Figure 5c shows M–H curves for the In_0.1_Ga_0.9_N, In_0.3_Ga_0.7_N and In_0.5_Ga_0.5_N nanostructures at 300 K. For the InN nanorods, Ms increases as the diameter of the nanostructure increases. This should also be effective in InGaN nanostructures. However, the In_0.1_Ga_0.9_N nanorods with the smallest diameter have the largest Ms and Hc. The experimental results show that at the same dose of Cr^+^, Ms varies greatly with indium concentration, especially for the samples with an indium concentration of 10%. Considering that the nanorods are not perfectly perpendicular to the substrate, we measured out-of-plane loops on In_0.1_Ga_0.9_N nanorods (see supplementary Figure S1), the results of in-plane and out-of-plane are close. In addition, considering the inconsistency of the nanorod size, we recalculated the data according to the nanorod size (see supplementary Figure S2), and the conclusion did not change. Therefore, the concentration of indium, that is, the variation of the band structure of the InGaN, is more important than the morphology of the Cr-doped nanostructures on the magnetic properties. We also measured the zero-field cooled (ZFC) and field-cooled (FC) magnetization curves for these nanostructures. Figure 5d shows a representative M–T curve of In_0.1_G_a0.9_N nanorods measured at H = 500 Oe. There is no significant separation between the ZFC and FC curves. The smooth and featureless M–T curve indicates that there are no parasitic magnetic phases.

## 3. Materials and Methods

InN thin films and nanorods were prepared on 3 μm thick GaN (0002) substrates by using the radio-frequency plasma-assisted molecular beam epitaxy (RF-MBE, SVTA 35-V-2, SVT Associates, Inc., Eden Prairie, MN, USA). The GaN substrates were thermally cleaned at 800 °C for 10 min to remove the surface contaminants. InN nanorods were directly grown on the GaN substrate at 500 °C. InN thin films with a thickness of approximately 600 nm were deposited on a GaN buffer layer grown at 400 °C. The N_2_ flow rate and RF-plasma power were set at 2.5 standard cubic centimeters per minute (sccm) and 375 W throughout the growth procedures. In and Ga cell temperatures were set at 760 °C and 940 °C, respectively. InGaN nanorods were grown on thermally cleaned Si (111) substrates with a 30 nm AlN buffer layer. The size and density of InGaN nanorods were controlled by adjusting the element ratio, growth time, and growth temperature of the AlN buffer layer. The growth process was monitored by the reflection high-energy electron diffraction (RHEED, SVT Associates, Inc., Eden Prairie, MN, USA).

Before the implantation, all the samples were ultrasonically cleaned in the acetone, ethanol, and deionized water for 15 min, and then, they were soaked into the hydrofluoric acid (5%) to remove the oxide layers. The depth distribution of Cr^+^ was calculated by the SRIM 2013 program. The Cr^+^ implantation was conducted by using an ion implanter (LC22-100-01, Beijing Zhongkexin Electronics Equipment Co., Ltd., Beijing, China) at an accelerating voltage of 110 keV with doses of 2.6 × 10^15^, 5.3 × 10^15^, and 1.3 × 10^16^ ions/cm^2^. The implantation voltage and dose were based on SRIM simulations. The implantation direction was perpendicular to the sample surface. The project range of 110 keV Cr^+^ was located at about 50 nm below the surfaces. According to the number of ions within the range of 50 nm, the calculated Cr doping concentration were 0.5%, 1.0%, and 2.5%, respectively.

Annealing was carried out for 30 s in flowing N_2_ in a rapid thermal annealing (RTA) furnace after the implantation. The annealing temperatures for InN and InGaN samples were set at 500 and 600 °C, respectively.

The samples were characterized by high-resolution X-ray diffraction (HR-XRD, Bede D1, Bede Co., Durham, UK) and field-emission scanning electron microscope (FESEM, Versa 3D, FEI Company, Hillsboro, OR, USA). The magnetic properties were studied with a vibrating sample magnetometer (VSM) equipped in a physical property measurement system (PPMS-9, Quantum Design, Inc., San Diego, CA, USA). The magnetization loops were recorded with the magnetic field from −20,000 Oe to 20,000 Oe applied parallel to the samples’ surfaces. The unit of magnetic moment is emu in raw data. We convert emu to emu/cm^3^ to calculate Ms. The height of the nanorods is around 600 nm, and the thickness of the thin films is also around 600 nm. Considering that only the Cr-doped part is ferromagnetic, we set the sample height to 120 nm. Then, the volume of the sample is 5 mm × 2 mm × 120 nm. Ms = emu × 10^7^/(5 × 2 × 1.2) cm^−3^. The valence state of Cr was characterized by X-ray photoelectron spectroscopy (XPS, ESCALAB250Xi, Thermal Fisher Scientific, Waltham, MA, USA). Photoluminescence (PL, LabRAM HR800, HORIBA, Paris, France) measurements were conducted at the room temperature in the wavelength range of 350–700 nm and 600–1000 nm to analyze the optical properties of the InGaN nanostructures.

## 4. Conclusions

In_x_Ga_1−x_N (x = 0.1, 0.3, 0.5 and 1.0) nanostructures were prepared by MBE. The diamagnetic nanostructures were transformed into room temperature ferromagnetic after Cr^+^ implantation followed by annealing. Ms and Hc increase with the increasing Cr^+^ dose. The effect of Cr doping on ferromagnetism is greater than that caused by the implantation-induced defects. The Ms of the InN nanorods with diameters of 100–160 nm is larger than that of the InN nanorods with diameters of 60–80 nm. The InGaN nanorods with a higher indium concentration have larger diameters. However, the Ms of the InGaN nanorods with 10% indium concentration reaches the largest, implying the most important factor of band energy regulation on the magnetic properties.

## Supplementary Materials

The following are available online at https://www.mdpi.com/2079-4991/10/6/1128/s1, Figure S1: (a) In-plane and out-of-plane loops for In_0.1_Ga_0.9_N nanorods; (b) Loops at lower fields, Figure S2: (a) M–H curves for the In_0.1_Ga_0.9_N, In_0.3_Ga_0.7_N and In_0.5_Ga_05_N; (b) M–H curves for the In_0.1_Ga_0.9_N (Eq), In_0.3_Ga_0.7_N (Eq), and In_0.5_Ga_0.5_N (Eq).
