# Supplementary material for: Room Temperature Ferromagnetism in InGaN Nanostructures Induced by Cr+ ion Implantation
Source: Nanomaterials (Basel). 2020 Jun 8;10(6):1128. doi: 10.3390/nano10061128 (PMC7353168; doi:10.3390/nano10061128)
Supplement: Supplementary file 1 [file nanomaterials-10-01128-s001.pdf]

Supplementary Material

Room Temperature Ferromagnetism in InGaN Nanostructures Induced by Cr^+ ion Implantation

Zheng Wang [1], Hao Wu [2,*], Yong Liu [2] and Chang Liu [1,*]

[1] Key Laboratory of Artificial Micro- and Nano-structures of Ministry of Education, and School of Physics and Technology, Wuhan University, Wuhan 430072, China; wz@whu.edu.cn
[2] Hubei Nuclear Solid Physics Key Laboratory, and School of Physics and Technology, Wuhan University, Wuhan 430072, China; yongliu@whu.edu.cn
* Correspondence: h.wu@whu.edu.cn (H.W.); chang.liu@whu.edu.cn (C.L.)

The rods are not perfectly perpendicular to the substrate. The easy axes of different rods will probably be oriented in different directions, hence, affecting Hc. We measured out-of-plane loops on $In_{0.1}Ga_{0.9}N$ nanorods, the results of in-plane and out-of-plane are close.

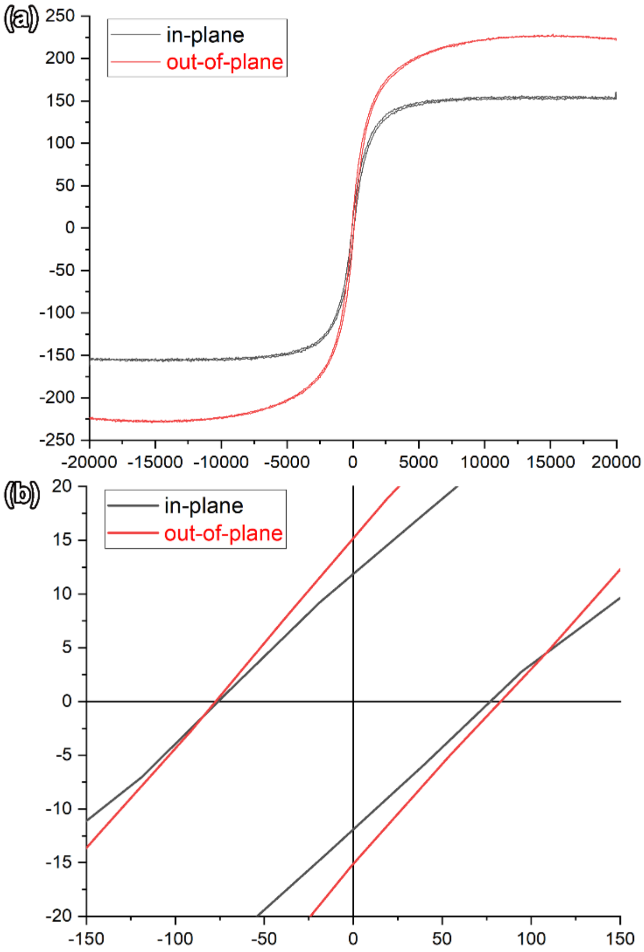

Figure S1. (**a**) In-plane and out-of-plane loops for the $In_{0.1}Ga_{0.9}N$ nanorods; (**b**) Loops at lower fields.

The size of the rods of some of the samples is rather inhomogeneous. By calculating the gray histogram of the FESEM image, the diameter distribution and area of the nanorods can be obtained with an error of 10%. We define this equivalent Ms as Ms (Eq). Ms (Eq) = Ms (nanorods) / area (nanorods). After recalculation, Ms becomes larger, Hc changes very little. The conclusion has not changed, indium concentration is still the most important factor.

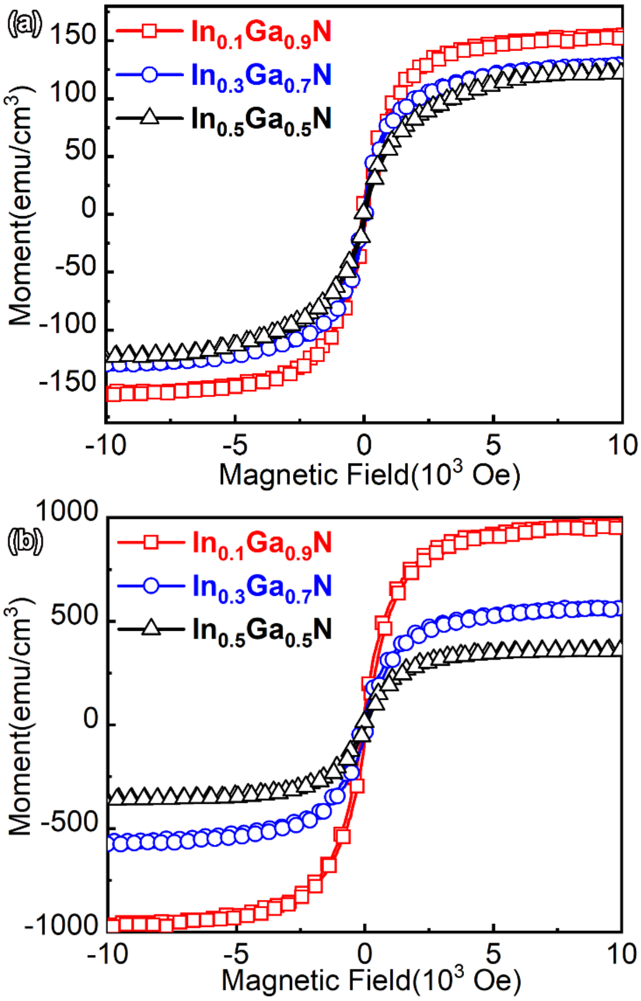

Figure S2. (a) M–H curves for the $In_{0.1}Ga_{0.9}N$, $In_{0.3}Ga_{0.7}N$ and $In_{0.5}Ga_{0.5}N$. (b) M–H curves for the $In_{0.1}Ga_{0.9}N$ (Eq), $In_{0.3}Ga_{0.7}N$ (Eq), and $In_{0.5}Ga_{0.5}N$ (Eq).

In order to eliminate the influence of inconsistency of nanorods, we have done a lot of control groups, such as the case where the inter-rod spacing is approximate, but the rod diameters are different; the same indium concentration, but the inter-rod spacing varies greatly. However, the difference in Hc in these cases is not as obvious and regular as the change in Hc when indium concentration is changed.
